# Supplementary material for: Development and validation of the Japanese Moral Foundations Dictionary
Source: PLoS One. 2019 Mar 25;14(3):e0213343. doi: 10.1371/journal.pone.0213343 (PMC6433225; doi:10.1371/journal.pone.0213343)
Supplement: S1 Text — (PDF) [file pone.0213343.s001.pdf]

S1 Text. Brief explanations of Haidt's five moral foundations.

The descriptions of the five moral foundations presented to participants were concise and brief in order to use as few dictionary words as possible:

<u>Harm</u>
Morality of no harm: This morality is concerned with protecting the weaker. You should not offend others physically or mentally.

<u>Fairness</u>
Morality of fairness: You should not do something unfair, cheat, or lie.

<u>Ingroup</u>
Morality of fellowship: You should take care of your fellows. To fulfill your role in your group, you should not betray your fellows.

<u>Authority</u>
Morality of respect: You should respect the elderly and the hierarchy of relationships.

<u>Purity</u>
Morality of cleanliness: You should avoid anything that is dirty or that degrades the sacred.

All the descriptions were followed by the instructions below:
Please freely describe as many situations (events/actions) as possible where this moral ethic is violated according to what you have witnessed, experienced, or can imagine.
Please write at least one situation.
