# Supplementary figures and images for: Development and validation of the Japanese Moral Foundations Dictionary
Source: PLoS One. 2019 Mar 25;14(3):e0213343. doi: 10.1371/journal.pone.0213343 (PMC6433225; doi:10.1371/journal.pone.0213343)

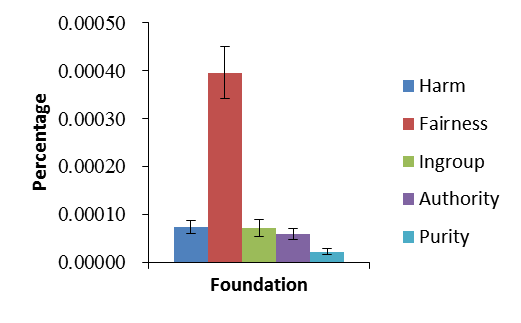

Supplement: S1 Fig — (TIF) [file pone.0213343.s002.tif]

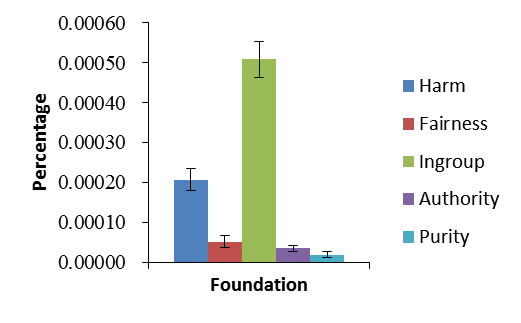

Supplement: S2 Fig — (TIF) [file pone.0213343.s003.tif]

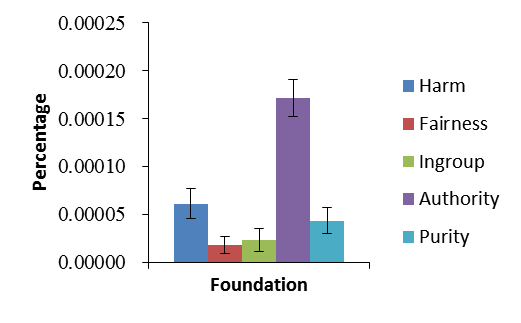

Supplement: S3 Fig — (TIF) [file pone.0213343.s004.tif]

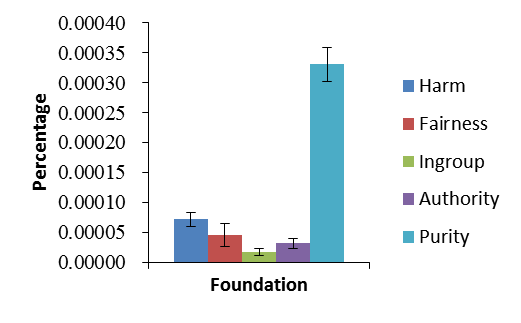

Supplement: S4 Fig — (TIF) [file pone.0213343.s005.tif]
